# Supplementary material for: Resolving the full spectrum of human genome variation using Linked-Reads
Source: Genome Res. 2019 Apr;29(4):635–45. doi: 10.1101/gr.234443.118 (PMC6442396; doi:10.1101/gr.234443.118)
Supplement: Supplemental Material [file supp_gr.234443.118_Supplemental_longranger-2.2.zip › longranger-2.2.tar.bz2!/lr2.2-public/lib/stan/cmdstan/stan/doc/api/html/structstan_1_1mcmc_1_1nuts__util.html]

Stan: stan::mcmc::nuts\_util Struct Reference


|  |  |
| --- | --- |
|  | Stan  2.5.0  probability, sampling & optimization |


- Main Page
- Related Pages
- Namespaces
- Classes
- Files

- Class List
- Class Index
- Class Hierarchy
- Class Members

All Classes Namespaces Files Functions Variables Typedefs Enumerations Enumerator Friends Macros Pages

- stan
- mcmc
- nuts\_util

Public Attributes |
List of all members

stan::mcmc::nuts\_util Struct Reference

`#include <base_nuts.hpp>`

|  |  |
| --- | --- |
| Public Attributes | |
| double | log\_u |
|  | |
| double | H0 |
|  | |
| int | sign |
|  | |
| int | n\_tree |
|  | |
| double | sum\_prob |
|  | |
| bool | criterion |
|  | |

## Detailed Description

Definition at line 14 of file base\_nuts.hpp.

## Member Data Documentation

bool stan::mcmc::nuts\_util::criterion

Definition at line 24 of file base\_nuts.hpp.

double stan::mcmc::nuts\_util::H0

Definition at line 18 of file base\_nuts.hpp.

double stan::mcmc::nuts\_util::log\_u

Definition at line 17 of file base\_nuts.hpp.

int stan::mcmc::nuts\_util::n\_tree

Definition at line 22 of file base\_nuts.hpp.

int stan::mcmc::nuts\_util::sign

Definition at line 19 of file base\_nuts.hpp.

double stan::mcmc::nuts\_util::sum\_prob

Definition at line 23 of file base\_nuts.hpp.

---

The documentation for this struct was generated from the following file:

- src/stan/mcmc/hmc/nuts/base\_nuts.hpp


---

[ Stan Home Page ]

*© 2011–2014,
Stan Development Team.*
